# Genetic Analysis of Fibroblast Growth Factor Signaling in the *Drosophila* Eye

**DOI:** 10.1534/g3.111.001495

**Published:** 2012-01-01

**Authors:** T. Mukherjee, I. Choi, Utpal Banerjee

**Affiliations:** * Department of Molecular, Cell and Developmental Biology and; † Davis School of Gerontology, Ethel Percy Andrus Gerontology Center, University of Southern California, Los Angeles, California 90089-0191, and; ‡ Molecular Biology Institute; § Department of Biological Chemistry; ** Eli and Edythe Broad Center of Regenerative Medicine and Stem Cell Research, University of California, Los Angeles, California 90095

**Keywords:** cellular adhesion, cadherins, fibroblast growth factor (FGF) signaling, morphogenetic furrow, glial cell migration

## Abstract

The development of eyes in *Drosophila* involves intricate epithelial reorganization events for accurate positioning of cells and proper formation and organization of ommatidial clusters. We demonstrate that Branchless (Bnl), the fibroblast growth factor ligand, regulates restructuring events in the eye disc primordium from as early as the emergence of clusters from a morphogenetic front to the cellular movements during pupal eye development. Breathless (Btl) functions as the fibroblast growth factor receptor to mediate Bnl signal, and together they regulate expression of DE-cadherin, Crumbs, and Actin. In addition, in the eye Bnl regulates the temporal onset and extent of retinal basal glial cell migration by activating Btl in the glia. We hypothesized that the Bnl functions in the eye are Hedgehog dependent and represent novel aspects of Bnl signaling not explored previously.

The fibroblast growth factor (FGF) signaling pathway elicits a wide range of cellular processes ranging from mitogenesis, angiogenesis, cell proliferation, differentiation, and migration (Powers *et al.* 2000). Three FGF ligands (Pyramus, Thisbe, and Bnl) and two FGF receptors (Heartless [Htl] and Btl) (reviewed by Ornitz and Itoh 2001; Szebenyi and Fallon 1999) have been identified in *Drosophila*. Bnl functions as a chemoattractant to guide branch budding and outgrowth of the Btl-expressing tracheal cells (Klambt *et al.* 1992; Sutherland *et al.* 1996). Htl is essential for mesodermal patterning (Gisselbrecht *et al.* 1996) and during migration and morphogenesis of interface glial cells (Shishido *et al.* 1997). The ligands Pyramus and Thisbe mediate Htl function (Kadam *et al.* 2009). In *Caenorhabditis* *elegans*, the only FGF receptor, EGL-15, allows migration of sex myoblasts. In vertebrates, FGF promotes branching morphogenesis (Peters *et al.* 1994). FGF4 and FGF8 are essential for cell migration (reviewed in Huang and Stern 2005).

Cellular adhesion plays an integral role during migration as cells constantly replenish old junctions with new ones. During development of the *Drosophila* eye disc, a precise coordination of morphogenetic processes with other cellular events promotes proper patterning. Here, we show that FGF controls morphogenetic movements during larval and pupal *Drosophila* eye development by maintaining cellular adhesion and adherens junction (AJ) proteins. In addition, we demonstrate that Bnl from the eye controls proper migration of the retinal basal glial (RBG) cells into the eye disc.

Photoreceptor cells in the eye are born as a wave of differentiation called the morphogenetic furrow (MF) sweeps across the eye from the posterior to the anterior. The RBG cells originate from precursors in the optic stalk and migrate into the eye disc. This is closely coordinated with the onset of photoreceptor differentiation, and the RBGs terminate migration 3rd to 4th ommatidial columns posterior to the MF (Choi and Benzer 1994; Rangarajan *et al.* 1999; Silies *et al.* 2007). Pyramus, expressed by glia, stimulates glial proliferation and motility, whereas neuronally expressed Thisbe induces glial differentiation and terminates migration (Franzdottir *et al.* 2009). Htl is involved in mediating these functions (Franzdottir *et al.* 2009). Here we show nonautonomous Bnl/Btl signaling regulates RBG migration, where the RBGs express Btl and sense Bnl from the eye. Overall, this study indicates a coordinated role for Bnl/Btl signaling in eliciting differential responses during multiple stages of eye development determined by cell autonomy, given its requirement for proper formation of clusters in the larval eye to maintaining tissue architecture in pupal eye discs and its requirement during RBG migration.

## Materials and Methods

The following fly stocks were used: *y w ey-flp*; *FRT82B Ubi-GFP RpS3/TM6B Tb*, *y^+^*, *y w ey-flp*; *FRT80B Ubi-GFP RpS3/TM6B Tb*, *y^+^*, *y w ey-flp*; *ey-GAL4*; *FRT80B Ubi-GFP RpS3/TM6B Tb*, *y^+^*, *P{dpp-lacZ}*, *y w*; *FRT80B btl^LG19^/TM6B Tb*, *y^+^*, *y w*; *FRT82B bnl^00857^/TM6B Tb*, *y^+^*, *hh^ts2^*, *UAS-cdc42^N17^*, *UAS-λbtl*, and *UAS-λhtl* (Bloomington), *y w*; *FRT82B bnl^JR69^/TM6B Tb*, *y^+^* (T. Liao), *w*;*UAS* > *CD2* > *y*+>*mCD8GFP/CyO*; *repo-GAL4*, *UAS-FLP/Tb* (C. Klaembt). RNAi stocks: *UAS-btl^RNAi^*, *UAS-bnl^RNAi^*, *UAS-dof^RNAi^*, and *UAS-htl^RNAi^* (Vienna Drosophila Rnai Center [VDRC], Vienna, Austria).

For immunohistochemical analysis, the following primary antibodies were used: rat anti-BrdU (1:100; Abcam, Cambridge, MA), rabbit anti-cleaved Caspase 3 (1:500; Cell Signaling Technology, Beverly, MA), rat anti-Bnl (1:50; M. Krasnow), rat anti-Shg (1:500; V. Hartenstein), mouse anti-Dlg (1:100; Developmental Studies Hybridoma Bank [DSHB], Iowa City, IA), mouse anti-Crumbs (1:200; DSHB), mouse anti-DN-cadherin (1:200; DSHB), mouse anti-Repo (1:10; DSHB) and mouse anti-Armadillo (1:100; DSHB). The secondary antibodies were obtained from Jackson ImmunoResearch.

Quantification of apical cell surfaces in the MF was performed with the use of a published protocol (Corrigall *et al.* 2007). Mean apical cell surface area (μm^2^)/cell for mutant and wild-type tissue are given. The 2-tailed Student’s paired *t*-test was applied to all the quantifications.

## Results and Discussion

### Bnl/Btl signaling during *Drosophila* eye development

**Figure 1:**
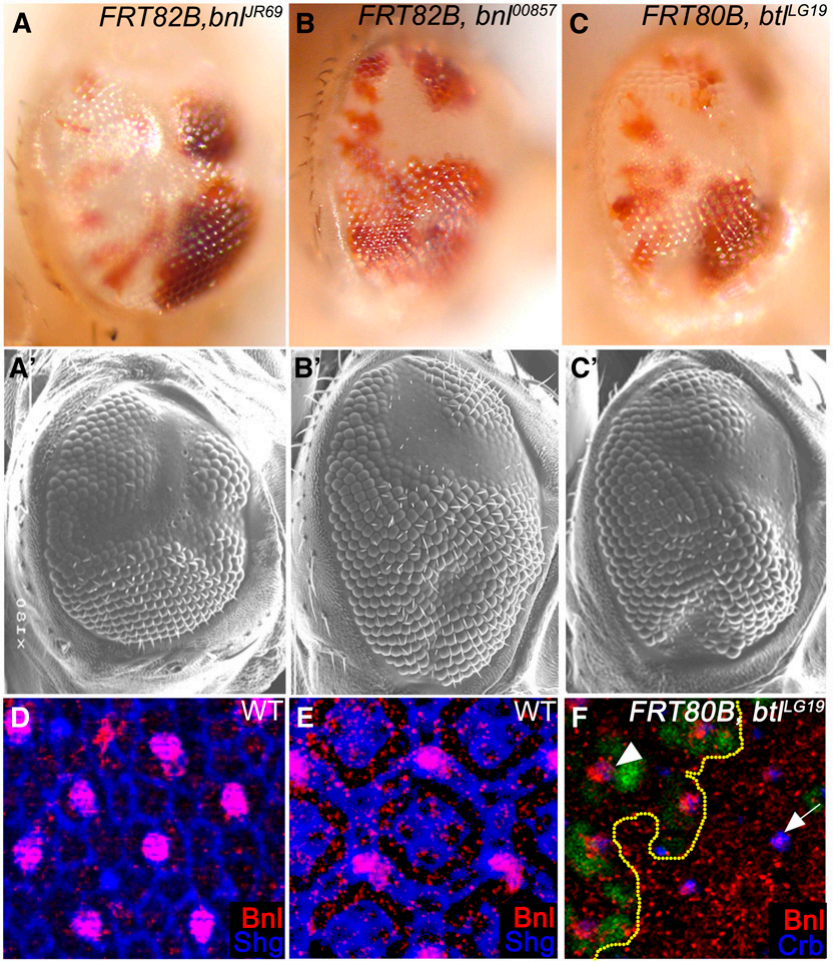
Bnl/Btl functions during *Drosophila* eye development. (A-C′) Loss of Bnl/Btl signaling affects eye development. Bright field images (A-C) containing somatic clones (marked by the absence of red pigmentation) and (A′-C′) the corresponding scanning electron micrographs with (A-A′) *bnl^JR69^/bnl^JR69^*, (B-B′) *bnl^00857^/ bnl^00857^*, and (C-C′) *btl^LG19^/btl^LG19^*. Wild-type facets are red and faceted, and the mutant tissue (white) appears glossy in both light and electron micrographs. Pupal eye disc stained with Bnl (red) protein shows elevated levels in (D) cone and (E) bristle cells, co-stained with anti-Shg (blue) antibody. (F) Somatic clones of *btl^LG19^* (nongreen tissue) in pupal eye discs have reduced Bnl protein (red) expression in the cone cells (marked using Crumbs in blue).

In a screen to identify mutations with patterning defects in the adult eye (Liao *et al.* 2006), we identified a loss-of-function allele of *bnl*, *bnl^JR69^* that gives a “glossy-eye” phenotype (Figure 1, A and A′). This new allele fails to complement *bnl^00857^*, a well-characterized loss-of-function allele of *bnl* (Jarecki *et al.* 1999; Sutherland *et al.* 1996). *bnl^00857^* clones in the eye also phenocopy the glossy-eye phenotype (Figure 1, B and B′). Loss of *btl* receptor function, induced by use of the *btl^LG19^* allele, (Klambt *et al.* 1992; Reichman-Fried *et al.* 1994; Sutherland *et al.* 1996) also results in a similar phenotype (Figure 1, C and C′), whereas loss *htl*, does not phenocopy this effect (not shown). This represents a novel function for Bnl-mediated Btl signaling during *Drosophila* eye development not explored previously. Immunohistochemical studies reveal uniform low expression of Bnl protein in 3rd instar eye disc (data not shown). Whereas in the pupal eye discs aged approximately 28 hrs after pupal formation (APF), Bnl expression is restricted to the cone cells (Figure 1D) and later (~48 hrs APF) detected in the bristle cells (Figure 1E). *btl^LG19^* clones in pupal eye disc (~28 hrs APF) have reduced Bnl protein expression (Figure 1F) residual, mislocalized staining is seen away from the tips of the cone cells, suggesting that ligand-receptor interaction promotes proper localization and stabilization of Bnl.

### Bnl signaling regulates cellular adhesion during *Drosophila* eye development

**Figure 2:**
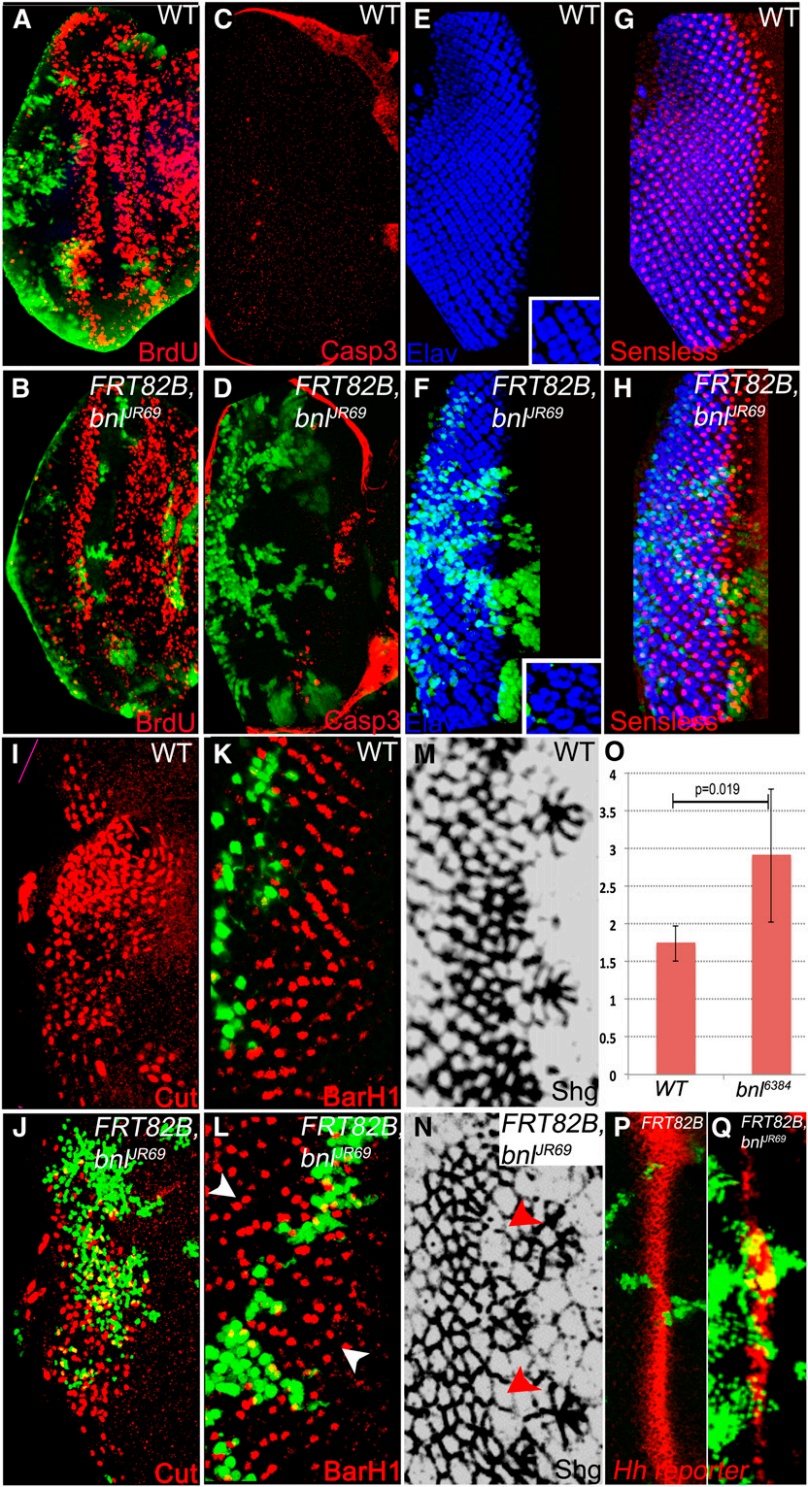
Bnl signaling regulates cell shape changes in the furrow. In all panels, eye discs from 3rd instar larvae are shown. Posterior is to the left. (A-B) Incorporation of 5-bromo-2′-deoxyuridine (BrdU; red) marks cells in S phase in 3rd instar larval eye disc. (A) Normal pattern of BrdU incorporation, where both the green and nongreen tissues are wild type. (B) Somatic clones of *bnl^JR69^* (nongreen) show no defect in BrdU incorporation. (C-D) Assay for cell death using anti-cleaved Caspase 3 (red) antibody. (C) Wild-type and (D) eye discs containing somatic clones of *bnl^JR69^* (non-green) show no difference in cell viability. (E-L) Loss of *bnl* function does not affect cell fate specification as visualized by expression of (E-F) Elav, marks photoreceptor neurons (blue), (G-H) Senseless is a marker for R8 (red), (I-J) Cut, marks cone cells (red), and (K-L) BarH1 is a marker for R1 and R6. Compare wild-type panels (E, G, I, and K) correspondingly with somatic clones of *bnl^JR69^* (nongreen, F, H, J, and L). *bnl^JR69^* clones show defects in ommatidial arrangement evident from organization of BarH1 expressing cells (L compared with K). Compared with (M) wild-type pattern of Shg (gray) expression at the MF, (N) *bnl^JR69^* somatic clones shows loss in ommatidial organization (arrowheads). (O) Graphical representation of the mean surface area (μm^2^)/cell (shown on the y-axis) of the cells within the MF from wild-type and *bnl^00857^* mutant clones (n = 6, *P* = 0.01). (P) Wild-type expression pattern of *Hh* reporter (red) is down-regulated (Q) in somatic clones of *bnl^JR69^* (nongreen).

To investigate the developmental phenotype underlying Bnl dysfunction, somatic clones of mutant tissue were analyzed. *bnl* clones do not exhibit any defect or delay in G1-S transition (Figure 2, A and B) and early viability of the cells is unaffected (Figure 2, C and D). Cell fate specification markers are expressed in mutant cells (Figure 2, E−L). However, defects in ommatidial organization are seen using BarH1 antibody marking R1 and R6 photoreceptors that are normally organized in a linear pattern (Figure 2, K and L).

The earliest events in cluster formation involve morphogenetic changes and constriction of apical cell surfaces (Escudero *et al.* 2007; Ready *et al.* 1976; Wolff and Ready 1991). *bnl* mutant cells lose their ability to constrict their apical surfaces (Figure 2, M−O; numerically, at the morphogenetic furrow, wild-type cell surface/*bnl* mutant cell surface = 0.599, *P* = 0.01) and lose normal contacts between cells and form aberrant-shaped clusters (Figure 2N). Expression of the Hh activity-dependent reporter (*dpp-lacZ*), seen at high levels in the wild-type MF, is strongly reduced in *bnl^JR69^* clones (Figure 2, P and Q), implying an interaction between Hh and FGF signaling in regulating cellular adhesion and morphogenesis during larval eye development. Hh signaling has been implicated in similar phenotypes and regulates Myosin II activity necessary for cellular rearrangement at the furrow (Corrigall *et al.* 2007, Escudero *et al.* 2007). It is therefore these early defects in proper rosette formation at the furrow (Ready *et al.* 1976; Wolff and Ready 1991) that are responsible for the improper organization of the ensuing cluster (Figure 2, M and N).

**Figure 3:**
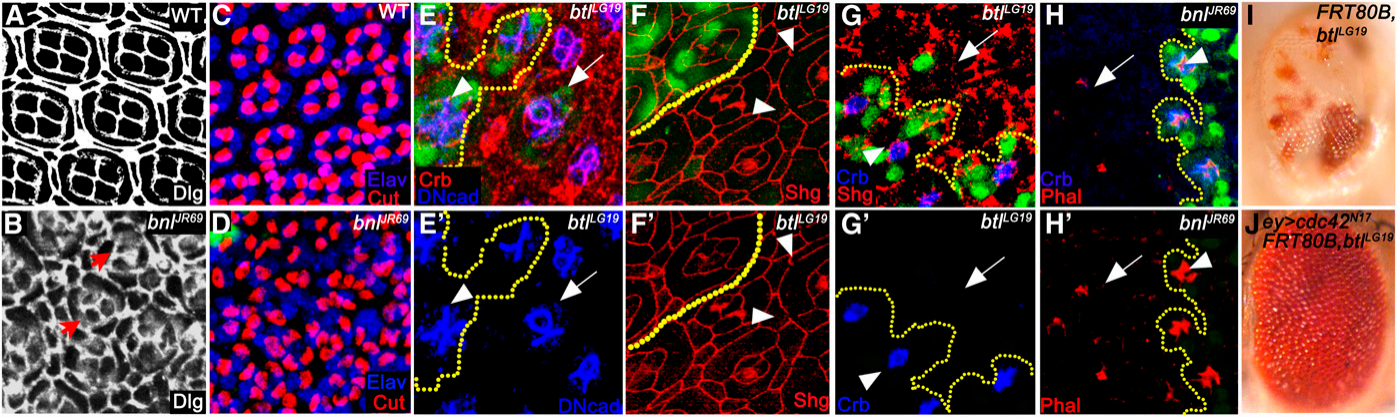
Bnl function maintains junctional proteins. In all panels, eye discs from pupal stages (48−50 hr APF) are shown. Compared with Dlg (gray) expression in wild type (A), *bnl^JR69^* clones (B) show defects in cluster organization (arrowheads). Elav (blue) and Cut (red) expression level in wild type (C) and *bnl^JR69^* (D) clones (nongreen) are comparable although cellular arrangement is disrupted in *bnl^JR69^* mutants. (E and E′) Compared with DN-cadherin (blue) and Crumbs (red) expression in wild-type tissue (green, arrowhead), *btl^LG19^* clones (nongreen) show defects in cone-cell architecture (arrow, n = 5). Compared with Shg (red) expression in wild-type tissue (F, green), *btl^LG19^* clones (F′, nongreen) show disrupted Shg expression at cell−cell junctions (arrowheads, n = 4). (G and G′) Crumbs (blue) and Shg (red) expression is lost from *btl^LG19^* mutant rhabdomere adherens junctions (AJ, nongreen, arrow, n = 5). Compare with surrounding wild-type rhabdomere AJ (green, arrowhead). (H and H′) Crumbs (blue) expression and phalloidin (red) staining are lost from *bnl^JR69^* mutant rhabdomere AJ (nongreen, arrow, n = 10). Compare with surrounding wild-type rhabdomere AJ (green, arrowhead). (I) *btl^LG19^* adult glossy-eye phenotype is rescued by (J) coexpressing a dominant-negative form of cdc42 (*cdc42^N17^*, n = 4).

The next set of morphogenetic defects caused by FGF signaling is evident during pupal development, where a series of coordinated and precise cell movements lead to a well-patterned pupal epithelium (Cagan 1993; Cagan and Ready 1989; Ready *et al.* 1976). This regularity in the wild-type pattern is evident upon staining with septate junction marker Discs large (Figure 3A) (Woods and Bryant 1991). In *bnl* mutants the architecture of the ommatidial clusters is distorted, and instead of the regular hexagonal arrays, they often appear circular (Figure 3B). In addition, the Discs large staining in these clones is cytoplasmic (Figure 3B) and not localized to the membrane as in wild type (Figure 3A). The architectural defect in *bnl* mutants is not attributable to loss in neuronal or non-neuronal differentiation (Figure 3, C and D). However, *bnl* mutant clusters often contain improper number of cells (Figure 3B). More specifically, staining for DN-cadherin normally expressed at the interface between wild-type cone cells (Hayashi and Carthew 2004) revealed reduced numbers and altered arrangement of cone cells in *btl^LG19^* mutant clusters (Figure 3, E and E′). The expression of Shotgun (DE-cadherin) in *btl* mutant clones is also fragmented and missing at several cell-cell contact points (Figure 3, F and F′). Crumbs protein, an apical marker found above AJs is observed at high levels in wild-type cells (Figure 3G) (Izaddoost *et al.* 2002). However, *btl* and *bnl* mutant tissues are devoid of Crumbs protein (Figure 3, G′ and H′) and they also lack Shotgun expression (Figure 3G′) and show defective actin organization (Figure 3H′). Small GTPases function downstream of many biological signals to regulate diverse cellular processes (Kuhn *et al.* 2000), including FGF (Wolf *et al.* 2002). We therefore investigated interactions with various activated and dominant-negative forms of small GTPases in *btl^LG19^* clones. A dominant-negative version of *cdc42* rescues the *btl^LG19^* glossy-eye phenotype (Figure 3, I and J), suggesting that cdc42 functions as a negative downstream effector of Bnl signaling. Overall, the data here strongly suggest a central role for autonomous FGF signaling in the eye for the maintenance of proper cell-cell contact, junction stability and cell shape changes during retinal development which is achieved by modulating the levels of key cell adhesion and junctional proteins: DE-cadherin, Crumbs, and actin.

### FGF signaling regulates RBG cell migration

**Figure 4:**
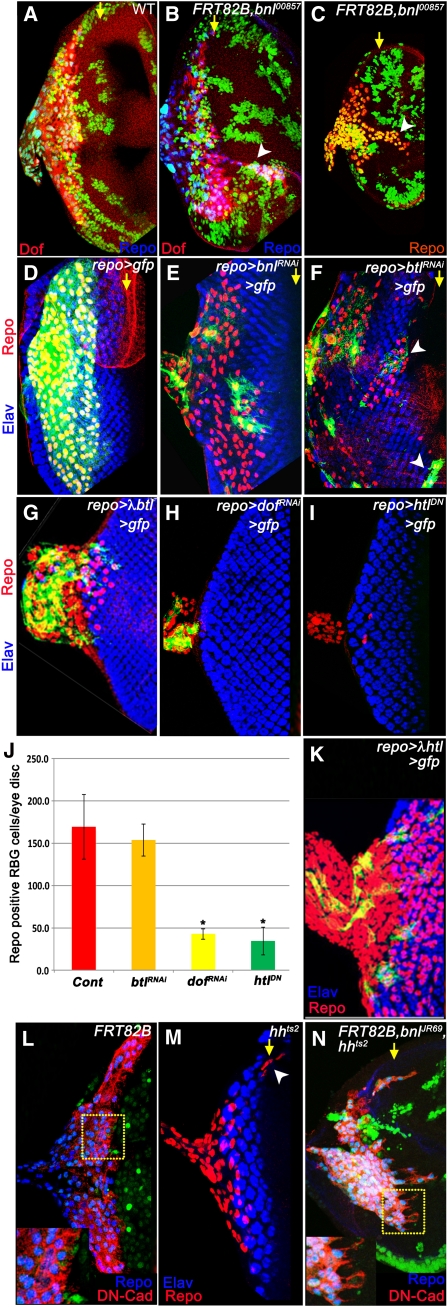
Bnl/Btl signaling regulates RBG migration. In all panels except panel C, eye discs are from wandering 3rd instar larvae. Posterior is to the left. In panel C, the eye disc is from a mid 2nd instar larva. Yellow arrows mark the MF. Compared with RBG (Repo: blue and Dof: red) in (A) wild-type (green and nongreen tissues are normal), (B) eye discs containing *bnl^00857^* clones have ectopically migrating RBGs (arrowhead B, compare with panel A). (C) 2nd instar eye discs containing *bnl^00857^* somatic clones (nongreen) show precocious RBG (Repo: red) migration. (D) Control eye disc expressing GFP in the RBG cells (green; *UAS > CD2 > y+>mCD8GFP/CyO; repo-GAL4*, *UAS-FLP/Tb; UAS-GFP*; Repo: red) costained with Elav (blue) to mark photoreceptor cluster. RBG cell (Repo: red) clones expressing (E) *bnl^RNAi^* (green; *UAS > CD2 > y+>mCD8GFP/CyO; repo-GAL4*, *UAS-FLP/Tb*; *UAS-bnl^RNAi^*) show no defect in glial migration, (F) *btl^RNAi^* (green; *UAS > CD2 > y+>mCD8GFP/CyO; repo-GAL4*, *UAS-FLP/Tb*; *UAS-btl^RNAi^*) causes ectopic RBG migration (marked by arrowheads, n = 7) and (G) constitutively active *btl* (green; *UAS > CD2 > y+>mCD8GFP/CyO; repo-GAL4*, *UAS-FLP/Tb*; *UAS-λ-btl*) inhibits RBG migration (n = 5). Elav (blue) marks the photoreceptor clusters. RBG cells (Repo: red) clones expressing (H) *dof^RNAi^* (green; *UAS > CD2 > y+>mCD8GFP/CyO; repo-GAL4*, *UAS-FLP/Tb; UAS-dof^RNAi^*) or (I) *htl^DN^* (*UAS > CD2 > y+>mCD8GFP/CyO; repo-GAL4*, *UAS-FLP/Tb; UAS-htl^DN^*) show reduction in RBG number. (J) Quantification of RBG cell counts from control (n = 12), *btl^RNAi^* (n = 12), *dof^RNAi^* (n = 5, * indicates *P* < 0.001) and *Htl^DN^* (n = 12, * indicates *P* < 0.001). (K) Expressing constitutively active *Htl* (green; *UAS > CD2 > y+>mCD8GFP/CyO; repo-GAL4*, *UAS-FLP/Tb; UAS-λ-Htl*) causes expansion of RBG cell numbers. Elav (blue) marks photoreceptor clusters. Compared to RBG cells (Repo: blue and DN-cadherin: red) in (L) wild type (green and nongreen tissue are normal), eye discs from (M) *hh* mutants and (N) containing *bnl^JR69^* and *hh* double mutant clones (nongreen) show ectopically migrating glia with long cytoplasmic extensions (small inset in N compared with control L).

In contrast to the tissue autonomous defects in patterning discussed previously, loss of *bnl* function in the eye also causes defects in RBG migration (Figure 4, A and B). In eye discs containing *bnl* mutant clones, the RBG cells migrate ectopically to anterior regions of the eye beyond the MF (Figure 4B), not seen in similarly aged wild-type controls (Figure 4A). This migration defect is observed as early as in 2nd instar eye discs lacking *bnl* function (Figure 4C), earlier than RBG migration in wild types. *bnl* clones generated specifically in the glia do not show RBG migration defects (Figure 4, D and E). In contrast, *btl^RNAi^* expressed in the RBG cells causes the ectopic glial migration phenotype (Figure 4F) and when a constitutively active form of the receptor, *λ-btl* is expressed in glial cells, their migration is completely impaired and the RBGs are restricted to the optic stalk (Figure 4G).

These results suggest that Bnl expressed by the eye tissue activates nonautonomous Btl signaling in the glial cells to regulate the temporal onset and extent of RBG migration. Of importance, loss of *bnl* or *btl* function did not affect RBG proliferation or survival (Figure 4J). When analyzed for R cell axon migration in *bnl* mutant clones, no obvious defect in their growth and projection was apparent (not shown). Downstream-of-FGF (Dof) functions as a positive effector of FGF signaling (Battersby *et al.* 2003) and is expressed at high levels in RBG cells (Rangarajan *et al.* 1999). As reported previously, *dof* mutants generated in the glia causes reduction in RBG cell number, loss of differentiation, and lack of migration (Figure 4, H and J) (Franzdottir *et al.* 2009). These phenotypes are strikingly different from that observed in *btl* mutants (Figure 4F).

Given that *htl* functions as a FGF receptor and can activate signaling via *dof*, *htl* mutant clones generated in the glia phenocopied all *dof* mutant phenotypes (Figure 4, I and J) and expression of a constitutively active form *htl* (*λ-htl*) in the glial cells resulted in extensive proliferation of the RBG cells, without affecting their migration (Figure 4K) (Franzdottir *et al.* 2009). Given the phenotypic differences between *bnl/btl* and *htl/dof* backgrounds, we conclude that *htl*-mediated *dof* function is required earlier in RBG development to regulate proliferation, differentiation, and ability to initiate migration, whereas *bnl/btl* signaling functions later to limit RBGs from migrating precociously into the eye disc. This could be achieved by either functioning as an antagonist to an attractive signal or by directly signaling to inhibit their migration. A noncanonical mechanism of Hh function in the eye also renders similar, albeit weaker, glial migration defects (Figure 4, L and M) as observed in *bnl* mutants (Hummel *et al.* 2002) and in addition to the loss of *hh* reporter in *bnl* mutant clones (Figure 2Q), Bnl functions either upstream or in parallel to Hh in regulating RBG migration. Interestingly, eye discs containing *hh* and *bnl* double-mutant clones show ectopically migrating RBG cells (Figure 4N) with long cytoplasmic extensions not seen in wild-type RBG (Figure 4L) as observed by DN-cadherin expression.

*Drosophila* compound eye formation, comprising neuronal and non-neuronal cells in the eye disc proper and glia at the basal layer, is achieved by closely coordinating multiple developmental events. An important process involved is the migration of the RBG cells into the eye disc efficiently synchronized with the onset of differentiation and movement of the MF. The glial cells do not outpace the furrow. The coordination between the two independent processes, the initiation of neuronal development and glial migration, is intriguing. This study establishes that a single pathway involving Bnl and Btl is implicated in the emergence of clusters from the MF in the larval eye, controls the formation and stability of AJ, and as well controls the temporal onset and extent of RBG migration. In addition, the ligands Pyramus and Thisbe are also important for glial development, where Pyrmaus/Htl activation promotes proliferation and motility and Thisbe/Htl activation inhibits migration and promotes differentiation (Franzdottir *et al.* 2009). It is interesting how the relatively simple FGF/FGF receptor system in *Drosophila*, operating in a cell at a specific time, can generate differential responses determined by cell autonomy and ligand/receptor combinations. This study further highlights the importance of sequential receptor tyrosine kinase activation to achieve coordinated migration of all glial cells, as abnormal glial migration is a feature of many human diseases.
